# Work‐related stress and coping methods of internists and primary care physicians during the COVID‐19 pandemic in Japan: A mixed‐method study

**DOI:** 10.1002/jgf2.560

**Published:** 2022-05-23

**Authors:** Kiyoshi Shikino, Akira Kuriyama, Michito Sadohara, Takahiro Matsuo, Kazuya Nagasaki, Yoshito Nishimura, Saori Nonaka, Masashi Izumiya, Mitsuru Moriya, Yoichi Ohtake, Tetsuya Makiishi

**Affiliations:** ^1^ Department of General Medicine Chiba University Hospital Chiba Japan; ^2^ Emergency and Critical Care Center Kurashiki Central Hospital Kurashiki Japan; ^3^ Department of Community, Family, and General Medicine Kumamoto University Hospital Kumamoto Japan; ^4^ Department of Infectious Diseases St. Luke's International Hospital Tokyo Japan; ^5^ Department of Internal Medicine Mito Kyodo General Hospital Mito Japan; ^6^ Department of General Medicine Okayama University Hospital Okayama Japan; ^7^ Minamisoma Municipal General Hospital Minamisoma Japan; ^8^ Department of Medical Education Studies, International Research Center for Medical Education, Graduate School of Medicine The University of Tokyo Bunkyo Japan; ^9^ Department of Psychosomatic Internal Medicine Health Sciences University of Hokkaido Hospital Sapporo Japan; ^10^ Department of Internal Medicine Imai Hospital Inagawa Japan; ^11^ Department of General Medicine, Faculty of Medicine Shimane University Izumo Japan

**Keywords:** burnout, COVID‐19, mixed method, stress coping, stress factor

## Abstract

**Background:**

The COVID‐19 pandemic has affected the mental health of health care workers. This study aimed to investigate the stress factors that cause burnout in Japanese physicians and their coping methods during the COVID‐19 pandemic.

**Methods:**

We conducted a sequential explanatory mixed‐method study to investigate the psychological responses of physicians in the early stages of the pandemic. A cross‐sectional, web‐based, anonymous survey was conducted among members of the American College of Physicians Japan Chapter to quantitatively investigate the stress factors and prevalence of burnout. An open‐ended questionnaire with questions about stress factors and coping methods was additionally administered. The qualitative data were analyzed using qualitative content analysis.

**Results:**

Among the 1173 physicians surveyed, 214 (18.2%) responded. Among the participants, 107 (50.0%) responded “yes” to the question “I feel or have felt very stressed at work during the COVID‐19 pandemic,” and 68 (31.8%) reported burnout symptoms. Those who reported feeling stress (117 respondents) were asked to select 12 items of the stress factors related to COVID‐19. The most significant stress factor related to COVID‐19 was “Perceived risk of spreading COVID‐19 to family members” (*n* = 47). Content analysis identified 12 categories for the stress factors and 7 for stress‐coping methods corresponding to COVID‐19 (Cohen's kappa = 0.84 and 0.95, respectively).

**Conclusion:**

Several distinct stressors existed during the COVID‐19 pandemic, which might be related to burnout among physicians. Practicing stress‐coping strategies, as identified in the present study, may help reduce work‐related stress and prevent burnout.

## Graphical abstract

In the COVID‐19‐related stress factor, a total of 12 categories and 39 subcategories were identified. ‘Material workload’, ‘Workplace relationship’, ‘Organisation’, ‘Achievement’, ‘Emotional workload’, ‘Human resource’, ‘Family’, ‘Infection control and prevention’, ‘Self‐care’, ‘Income’, ‘Infodemic’, and ‘Patient care’.

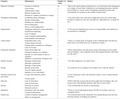

## 1 INTRODUCTION

The novel coronavirus disease (COVID‐19) was first identified in Wuhan, China, at the end of December 2019.^1^ Despite the dissemination of treatment and vaccines, the COVID‐19 pandemic continues to have a severe impact in different parts of the world. In Japan, the first COVID‐19 case was reported on January 16, 2020, and Japan was in the midst of the fourth wave of infection surge as of March 2021.^2^ Overall disease severity in the fourth wave was higher than that in the first through third waves. Internists and primary care physicians have played a central role as frontline healthcare workers in the diagnosis and treatment of COVID‐19 with continued uncertainty.^3^ The COVID‐19 pandemic has had detrimental effects on the mental health of healthcare workers, including burnout, anxiety, and depression.^3^, ^4^, ^5^, ^6^, ^7^, ^8^, ^9^, ^10^

Burnout is a syndrome characterized by emotional exhaustion, depersonalization, and a diminished sense of personal achievement.^11^ Burnout occurs in response to prolonged exposure to specific job demands when a person can no longer endure the stress they have been experiencing.^12^ Additionally, both quantity and quality of healthcare providers' care could be negatively affected by stress and burnout. Burnout is also known to lead to adverse outcomes in quality of patient care and increased health care costs.^13^, ^14^ Despite the significance of burnout as a public health issue, the stress factors and coping methods related to COVID‐19 have not been well investigated in the wake of COVID‐19.^15^

To reduce physician burnout, we need to know the stress factors that cause physician burnout and coping methods during the COVID‐19 pandemic. Naturally, burnout also exists in the non‐COVID‐19 period, but it is more apparent during the COVID‐19 pandemic. We also investigated the stress‐coping mechanisms physicians use to minimize these stress factors.

## 2 METHODS

### 2.1 Study design overview

Using a pragmatic approach, we used a mixed‐method design that incorporated quantitative (questionnaires) and qualitative (open‐ended questionnaire) techniques.^16^ We conducted a cross‐sectional, web‐based, anonymous survey of physician members of the American College of Physicians Japan Chapter (ACP‐JC) to quantitatively investigate the stress factors and prevalence of burnout. The ACP‐JC is mainly composed of internists and primary care physicians, including fellows, residents, and medical students. For this study, we asked only physicians working in Japan to respond to the survey. We sent an invitation to physician members on March 2, 2021, via email listservs of the ACP‐JC, which included a link to the survey on Google Forms. This period was the fourth wave of the surge with approximately 12,000 COVID‐19 hospitalizations patients in Japan.^17^ The fourth wave caused a medical crisis because beds for severe cases were at maximum capacity, restricting the transfer of some patients to advanced hospitals.^18^ Additionally, we sent three reminder emails, the last of which was sent on March 16, when we closed the survey. The survey was conducted between the end of the third wave of the COVID‐19 pandemic in Japan and another surge leading to the fourth wave. During this period, the number of newly diagnosed patients daily in Japan ranged from 922 to 1977, and the participants did not receive any incentives.

Qualitative data consisting of stress factors and stress‐coping methods based on internist and primary care physicians' perceptions were also collected. We aimed to conduct an exploratory study of stressors and stress‐coping during the COVID‐19 pandemic using quantitative and qualitative data.

### 2.2 Questionnaire design

The questionnaire variables were selected based on a literature review and discussion within the study group, which included internists, primary care physicians, psychiatrists, critical care physicians, and residents. The variables included were (1) personal information (age, sex, career duration, practice location, workplace, and marital status), (2) exposure to COVID‐19 (number of patients directly managed and infection of the participants and their families), (3) presence of stress factors related to the COVID‐19, (4) exacerbation of stress compared with the COVID‐19 period, and (5) Mini Z 2.0 Survey.^19^, ^20^

The Mini Z 2.0 Survey is a simple tool that can measure burnout and workplace conditions among physicians validated in Japanese.^20^, ^21^ We used the single‐item Mini Z Burnout Assessment (range 1–5) as follows^19^:

Please circle the option that best describes your situation based on your definition of “burnout”, and select one of the following response options:

1 = “You feel totally burned out. You are at a point where you may need some help.”

2 = “You always have symptom(s) of burnout. You are often worried about stress from work.”

3 = “You are beginning to burn out and have at least one symptom of burnout (e.g., emotional exhaustion).”

4 = “You feel under stress. You are not always full of energy but have never felt burned out.”

5 = “You enjoy working. You have never felt burned out.”

In the original version, respondents who choose option 3 (beginning of burnout) or lower are identified as having a burnout, which is the same in the Japanese version.

The scale has been validated with satisfactory correlation to the emotional subscale of the Maslach Burnout Inventory,^21^ a standard assessment for burnout. The presence of burnout was defined as a score of ≥3 on this item.

An open‐ended questionnaire was additionally administered with two questions: “Please tell us more about your stress and real‐life problems (stress factors)” and “Please tell us how you get help or handle the stress on your own (coping methods).” A qualitative inquiry was conducted following the quantitative study to better explain the quantitative results. Identifiers such as names were removed from the descriptions.^22^

The transcripts were analyzed using deductive content analysis, drawing upon the stress factors and stress‐coping methods as the coding frame with the cognitive process dimensions as the theme categories and subcategories.^23^, ^24^ Two authors (TM and KS) performed the open coding of stress factors, and two authors (MS and KS) performed the open coding of stress‐coping methods. They independently read and coded all the transcripts. They then discussed, identified, and agreed to the coding of the descriptors. The interrater degree of agreement between the researchers was assessed using Cohen's kappa statistic.^25^

The categories and subcategories derived by two authors (KN and YN) emerged from the data for triangulation in qualitative research.^26^ The categories and subcategories were regularly discussed and reviewed for content by an author (KS) with extensive experience in qualitative research to ensure the credibility of the findings.^26^ The concepts for each of the stress factors and stress‐coping methods were analyzed, and the number of units of analysis for each concept was counted.

Some research has focused on stress‐coping methods in physicians during non‐pandemic situations: problem‐focused strategies (active coping, instrumental support, and planning), emotion‐focused strategies (acceptance, emotional support, humor, positive reframing, and religion), and dysfunctional coping strategies (behavioral disengagement, denial, self‐distraction, self‐blame, substance use, and venting).^27^, ^28^, ^29^ The correspondence between these strategies and the subcategories created from the content analysis was examined.

### 2.3 Ethics approval and consent to participate

This study was approved by the Institutional Review Board of Kurashiki Central Hospital. We adhered to the consensus‐based checklist for reporting survey studies.^30^

### 2.4 Outcome measures

The primary outcome of the study was the prevalence of burnout and the percentage of COVID‐19‐related stress factors.

### 2.5 Statistical analysis

There were no missing data for any item. All statistical analyses were performed using SPSS version 26.0 (IBM Corp.).

## 3 RESULTS

Among the 1173 ACP‐JC physicians, 214 (18.2%) completed the survey. Table 1 provides the demographic details and other characteristics of the respondents. Among the participants, 107 (50.0%) responded “yes” to the question “I feel or have felt very stressed at work during COVID‐19 pandemic,” and 68 (31.8%) reported symptoms of burnout. Sixty‐one (28.5%) noted that their subjective level of burnout was worse compared to the pre‐COVID‐19 pandemic period. For the 107 participants who felt stressed at work related to the COVID‐19, the prevalence of burnout was 53.3% (*n* = 57), and 45.8% (*n* = 49) reported more burnout than in the pre‐COVID‐19 pandemic.

**TABLE 1:** Characteristics of the participants

Number of participants	214
Age, median (IQR)	54 (44–59)
Female, *n* (%)	24 (11.2%)
Career duration (years), *n* (%)	
1–5	13 (6.0%)
6–15	31 (14.5%)
16–25	47 (22.0%)
26–35	74 (34.6%)
≥36	49 (22.9%)
Practice location, *n* (%)	
Urban	123 (57.5%)
Suburban	52 (24.3%)
Rural	39 (18.2%)
Workplace, *n* (%)	
Clinic	48 (22.4%)
Community hospital less than 400 beds	65 (30.4%)
Community hospital over 400 beds	53 (24.8%)
University hospital	48 (22.4%)
Marital status, *n* (%)	
Married	180 (84.2%)
Divorced	5 (2.3%)
Single with a partner	6 (2.8%)
Single without a partner	21 (9.8%)
Widowed	2 (0.9%)
Total number of COVID‐19 patients managed, *n* (%)	
0	65 (30.4%)
1–30	124 (57.9%)
31–60	11 (5.2%)
≥61	14 (6.5%)
Burnout, *n* (%)	68 (31.8%)
Presence of stress factors related to the COVID‐19, *n* (%)	107 (50.0%)
Exacerbation of stress compared with the pre‐COVID‐19 period, *n* (%)	61 (28.5%)

Abbreviations: COVID‐19, coronavirus disease 2019; IQR, interquartile range.

**FIGURE 1:**
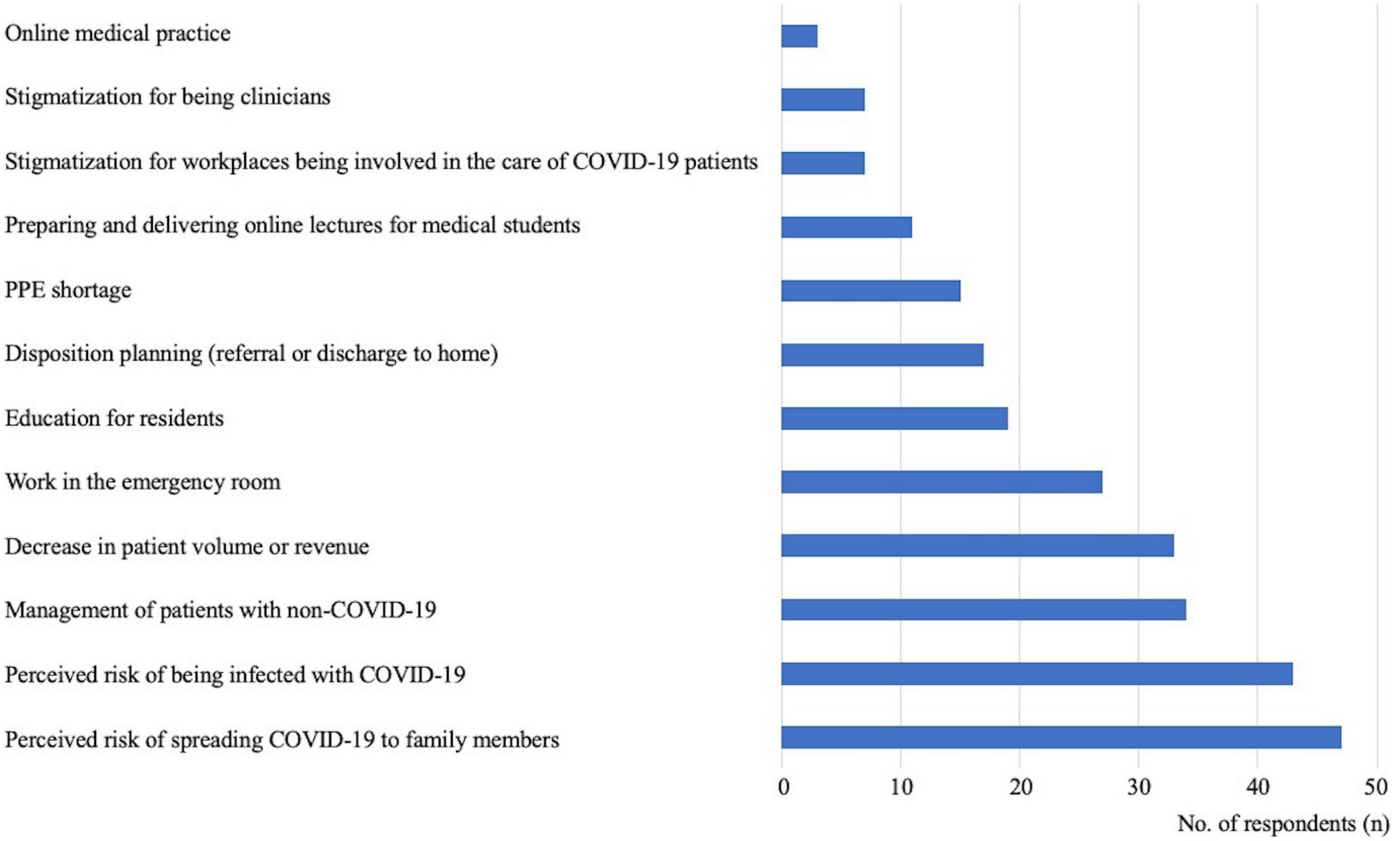
Respondents who reported feeling stress related to COVID‐19 were asked to select 12 related stress factors with the most common response being “Perceived risk of spreading COVID‐19 to family members” (*n* = 47)

In total, 107 respondents who reported feeling stress related to COVID‐19 were asked to select 12 items of the stress factors related to COVID‐19 (Figure 1). The most common responses were “Perceived risk of spreading COVID‐19 to family members” (*n* = 47).

### 3.1 Content analysis

A total of 84 participants responded to the open‐ended questions “Please tell us more about your stress and real‐life problems (stress factors)” and “Please tell us how you get help or handle the stress on your own (coping methods).” Stress factors and coping methods were explored using the obtained data. Following open coding, similar codes were grouped into categories. Categorical saturation was reached after analyzing the transcripts from the open‐ended questionnaires.

The absolute frequencies of the codes for stress factors and stress‐coping methods in the data of the present study are presented in Tables 2 and 3. In the COVID‐19‐related stress factor, a total of 12 categories and 39 subcategories were identified: “Material workload,” “Workplace relationship,” “Organization,” “Achievement,” “Emotional workload,” “Human resource,” “Family,” “Infection control and prevention,” “Self‐care,” “Income,” “Infodemic,” and “Patient care” (Table 2). A total of 7 categories and 49 subcategories were identified in the stress‐coping methods: “Self‐care activity,” “Mindset,” “Spending time with family,” “Physical activity,” “Sharing emotions,” “Communication,” and “Teaming” (Table 3). The most frequent subcategory by the number of codes was “Excessive workload” in the COVID‐19‐related stress factors and “Conversation with the family” in the stress‐coping method. The interrater reliability was substantial (Cohen's kappa = 0.84 and 0.95, respectively).

**TABLE 2:** Content analysis of COVID‐19‐related stress factors

Category	Subcategory	Number of codes	Quotes
Material workload	Excessive workload	10	“Due to the small number of physicians, even the head of the department is in charge of more than 10 patients as an attending physician, and this has resulted in a vicious cycle of inadequate education system and consequently no increase in the number of physicians.”
	Lack of time	2	
	Administrative tasks	1	
	Burden of paperwork	1	
	Increase in number of patients in charge	1	
Workplace relationship	Complaints about colleagues	3	“I am aware that I am in the most sought‐after job in the world today, but there is almost no one who can help me, understand me, or speak for me.”
	Human relationships	2	
	Building new relationships	1	
	Power harassment	1	
	Stress in staff employment	1	
Organization	Inadequate education system and policy	2	“I feel stressed regarding the exercise of responsibility and authority in my position as a manager.”
	Insufficient leadership	2	
	Lack of support	2	
	Lack of mutual understanding	1	
	Responsibility and authority	1	
Achievement	Suspended salary raise	2	“There is a strong sense of inequity in the workload and I feel that my hard work is not reflected in my evaluation or salary.”
	Anxiety about research	1	
	Feeling unfair compared to colleagues	1	
	Lack of rewards	1	
Emotional workload	Stress about night call	2	“With a full primary care physician system and being on call at night and on holidays, I am constantly tied to the hospital and have little time to truly relax.”
	Feeling of inequity in workload	1	
	Increase in work outside of area of expertise	1	
	Inability to concentrate on patient care	1	
Human resource	Shortage of physicians	3	“Too little manpower, too much work.”
	Shortage of generalist	1	
	Shortage of specialists	1	
Family	Burden on the child	1	“It is hard for me to continue to be unable to live with my family.”
	Care for elderly parents	1	
	Inability to live with family	1	
Infection control and prevention	Living with infection control	2	“As for living day to day with infection control, I feel a certain amount of stress.”
	Unclear goals	1	
Self‐care	Lack of relaxation	2	“With a full‐time system and on‐call at night and on holidays, they are always tied to the hospital and have little time to truly relax.”
	Lack of sleep time	1	
Income	Decrease in income	1	“I am worried that my income is decreasing.”
	Financial concern	1	
Infodemic	Frustration with the media	1	“There is a lot of information about COVID‐19 out there, and I am swamped with it.”
	Uncertain information	1	
Patient care	Conflict of patient management	1	“I'm stressed about all the situations I'm creating that prevent me from focusing entirely on helping my patients.”
	Delay in management	1	

**TABLE 3:** Content analysis of stress‐coping methods

Category	Subcategory	Number of codes	Quotes
Self‐care activity	Hobbies	3	“I am healed by my hobbies such as music and my dogs.”
	Pets	3	
	Watching TV and movies	3	
	Sleeping	2	
	Drinking	1	
	Enjoying short disposable time	1	
	Gaining more sleep time	1	
	Music	1	
	Reading	1	
	Regular bedtime	1	
	Securing the time not to be called	1	
	Singing	1	
	Taking a shower	1	
Mindset	Forgetting the job in extra time at work	2	“I try to forget about it within a short period of time because stress reduces motivation.”
	Asking oneself about dos and don'ts for job	1	
	Stop and think	1	
	Motivation by patient encouragement	1	
	Grit	1	
	I am what I am	1	
	Meditation	1	
	Not taking on alone	1	
	Probing	1	
	Reflection	1	
	Routine action	1	
	Taking not too serious	1	
	Inquiring	1	
	Thinking	1	
	Weighting more on the private life	1	
Spending time with family	Conversation with the family	7	“My stress relief is spending time with my family.”
	Spending time with family	3	
	Meal with the family	2	
	Complaining to family	1	
	Filtering the information	1	
	Supports from the family and friends	1	
	Work efficiency	1	
Physical activity	Exercise	5	“I calm my angry mood with exercise and running.”
	Running	2	
Sharing emotions	Let someone listen to me	1	“It's not “The King's Ear”, but…, I use closed social networking sites to let things out.”
	Chatting with outsiders	1	
	Closed social network service	1	
	Consultation with friends	1	
	Sharing information with colleagues	1	
	Talking	1	
Communication	Building relationships	1	“I'm coping with stress by talking to people around me.”
	Friendship	1	
	Friends' conversations	1	
Teaming	Assignment and split of the work	1	“We are all discussing and adjusting the overall workload and distribution.”
	Help from the admins	1	
	Support	1	

Of the 49 subcategories for stress‐coping methods related to COVID‐19, 14 were categorized as problem‐focused, 8 as emotion‐focused, and 27 as dysfunctional. Among the dysfunctional coping strategies, “Behavioral disengagement” was the most common (*n* = 15), followed by “Emotional support” (*n* = 7) among the emotion‐focused strategies. For problem‐focused strategies, there were “Active coping” (*n* = 4), “Instrumental support” (*n* = 4), and “Planning” (*n* = 5).

## 4 DISCUSSION

We extracted stress factors and stress‐coping methods among physicians during the COVID‐19 pandemic through a survey. Primarily, we investigate the psychological responses of physicians in the early stages of the pandemic using a mixed‐method approach. Previously investigated factors, such as the fear of getting infected or infecting family and friends, excessive workload, the intermittent shortage of personal protective equipment, and the need to take stressful precautions in the clinical setting and operative fields, can add enormous psychological burdens among frontline health care providers.^31^In this study, the most common stress factor related to COVID‐19 was the “Perceived risk of contamination of COVID‐19 to family members” (47/107 responses). In addition, content analysis extracted the same theme categories, such as “Material workload,” “Family,” and “Infection control and prevention,” and the relatively new themes categories such as “Income,” “Organization,” “Workplace relationship,” “Achievement,” “Human resource,” “Self‐care,” “Infodemic,” and “Patient care.”

Among these, one of the distinctive categories is “Infodemic.” The disease, combined with forced quarantine to combat COVID‐19 applied by nationwide lockdowns, can produce mental health problems.^32^ These have been fueled by an “Infodemic” spread via different social media platforms. Information about COVID‐19 is disseminated through media and social networking sites; however, there is also misleading and inaccurate information being disseminated, and such information overload can be significantly stressful.

The difficulty in using traditional methods of communication during the COVID‐19 pandemic is related to stress factors. One of the other categories is “Organization,” which includes the subcategory of “Insufficient leadership.” Emotional distress was associated with the experience of poor communication from supervisors,^33^ probably due to the scarcity of traditional communication. It may suggest that poor communication with the supervisor was one of the stress factors for physicians because they did not feel sufficient leadership from the supervisor.

For these stress factors, the content analysis extracted the following stress‐coping methods: “Self‐care activity,” “Mindset,” “Spending time with family,” “Physical activity,” “Sharing emotions,” “Communication,” and “Teaming.” One of the strengths of this survey is that it revealed stress‐coping methods during the COVID‐19 pandemic. The characteristics of these stress‐coping methods include rejuvenating through exercise, self‐care by getting enough sleep, and spending relaxing time with family and pets. In addition, sharing one's feelings with others and good communication with team members can be effective in coping with stress.

In the survey by the stress‐coping strategies subscale including 17 items for stress by COVID‐19, the following 7 items were used by all participants^34^: “Taking preventive measures,” “Actively learning about COVID‐19,” “Actively learning professional knowledge,” “Adjusting the attitude and facing the COVID‐19 epidemic positively,” “Chatting with families and friends,” “Recreational activities,” and “Engaging in health‐promoting activities.” The significant difference between these items was that the items listed defense and active learning against COVID‐19, which was not extracted in the current study. This was a different result from the data in the current study, in which Japanese internists and primary care physicians primarily used their leisure time, such as time with family, vacation, and mindset, as coping strategies. The differences may be due to some aspects of culture, the social system of medicine, and psychological reactions of those that have not yet accepted the pandemic.

When the results of the content analysis were compared for the three categories of problem‐focused, emotion‐focused, and dysfunctional coping strategies, dysfunctional coping strategies were the most common (*n* = 19). Dysfunctional coping strategies are not the preferred stress‐coping methods; however, during the COVID‐19 epidemic, people rely on stress‐coping that can be implemented to avoid infection, which may be why dysfunctional coping strategies were the most common. Another explanation was that participant physicians used self‐care and physical activities categorized in behavioral disengagement, distancing and avoiding the problem or work during the COVID‐19 pandemic. Even before the COVID‐19 pandemic, one mixed‐method study identified that physicians' coping strategies after leaving work were the use of exercise, having quiet time such as watching television, reading or mindless activities, and spending time with family.^35^ The next most common were problem‐focused strategies with a frequency of 14. Nevertheless, it is novel to find that positive stress‐coping methods, such as trying to improve the current situation and thinking about what should be done, are also actively used in the COVID‐19 pandemic.

The measures that can be taken at the organizational level to reduce stress factors in this way will be an issue for the future. It is essential to reduce these stressors and avoid burnout among Japanese internists and primary care physicians by using coping methods during the COVID‐19 epidemic. This study may be looking at psychological responses in the early stages of the COVID‐19 pandemic and may be generalizable to other pandemics and disasters.

### 4.1 Limitations

This study has several limitations. First, the response rates of our survey were low (18.2%). Previous web‐based unincentivized studies of burnout have reported a recovery rate of about 25%–31%.^36^, ^37^, ^38^ However, some evidence shows that response rates might be poorly associated with non‐responder bias.^39^ Considering that burnout is a sensitive topic and, further, the COVID‐19 pandemic, the low response rate may not have a significant impact on our survey. Second, according to the result of content analysis, most of the responses were about stress‐coping methods in individuals with fewer addressing organizational or system factors. This result may not have reached theoretical saturation regarding organization and systems. Stress‐coping methods associated with organizations and systems are also important and require further investigation. Third, the results of this survey may not be applicable to everyday stressors and stress‐coping methods internationally. Although Japanese physicians from various clinical settings and sites across Japan responded to the survey, the content may depend on Japan's unique culture. The external validity of this survey needs to be examined. Fourth, this quantitative survey was conducted only once, and no comparisons were made before or during the COVID‐19 pandemic. However, we added qualitative research and used a mixed‐method approach to augment the data. In particular, the qualitative studies have yielded an in‐depth review of internists' and primary care physicians' anguish and coping methods.

## 5 CONCLUSION

Several distinct stressors existed during the COVID‐19 pandemic that might be related to burnout among physicians. Practicing stress‐coping strategies, as identified in the present study, may help in reducing work‐related stress and preventing burnout.

## CONFLICT OF INTEREST

The authors have stated explicitly that there are no conflicts of interest in connection with this article.
